# Dapagliflozin, as Add-on Therapy in Type 2 Diabetes Patients, Is Associated With a Reduction in Albuminuria and Serum Transaminase Levels

**DOI:** 10.3389/fcdhc.2021.733693

**Published:** 2021-10-12

**Authors:** Silas Benjamin, Manjunath Ramanjaneya, Alexandra E. Butler, Imran Janjua, Firjeeth Paramba, Jafer Palaki, Aisha Al Kubaisi, Prem Chandra, Ibrahem Abdalhakam, Nasseer Ahmad Massodi

**Affiliations:** ^1^ Internal Medicine Department, Hamad Medical Corporation, Doha, Qatar; ^2^ Qatar Metabolic Institute, Hamad Medical Corporation, Doha, Qatar; ^3^ Research Department, Royal College of Surgeons in Ireland, Adliya, Bahrain; ^4^ Medical Research Center, Hamad Medical Corporation, Doha, Qatar

**Keywords:** diabetes mellitus type 2, dapagliflozin, albumin-creatinine ratio (ACR), glycosylated hemoglobin, alanine aminotransferase

## Abstract

**Introduction:**

SGLT-2 inhibitors are shown to be nephroprotective, slowing progression of nonalcoholic steatohepatitis (NASH) in addition to improving glycemic control in patients with type 2 diabetes (T2D). To date, no real-life clinical data is available on the effect of SGLT-2 inhibitors on urine albumin-creatinine ratio (ACR) and liver enzymes in a Middle Eastern population. Therefore, we evaluated the effect of dapagliflozin (DAPA) on urine ACR, alanine aminotransferase (ALT) and aspartate aminotransferase (AST) when added to standard therapy for T2D.

**Methods:**

This is an observational study of 40 patients with T2D in whom DAPA was added to their existing anti-diabetic regimen to improve glycemic control. The primary outcomes were changes in serum transaminase level and urine albumin-to-creatinine ratio (ACR). Secondary outcomes include changes in glycosylated hemoglobin (HbA1C), body mass index (BMI), oral hypoglycemic agents and insulin dose.

**Results:**

Whole group analysis showed a reduction in ALT (p<0.0001), (AST) (p=0.009), ACR (p=0.009) and BMI (p<0.0001) following DAPA treatment. Further sub-group analysis showed that patients on insulin and DAPA combination had a reduction in ACR (p=0.0090), ALT (p=0.0312), BMI (p=0.0007) and HbA1c (p<0.0001) compared to the sulfonylurea and DAPA combination group. In the sulfonylurea and DAPA combination group, there was a reduction in the sulfonylurea requirement following DAPA therapy (p=0.0116), with reductions in ALT (p=0.0122), AST (p=0.0362), BMI (p=0.0026) and HbA1c (p<0.0001) but with no change in ACR (p=0.814).

**Conclusion:**

In routine clinical practice, the addition of DAPA to standard medical therapy is well tolerated and beneficial for T2D patients and is associated with a reduction of ALT and ACR.

## Introduction

The relationship between diabetes and heart disease is well recognized and, as reported in the Framingham heart study, there is a ~2-3 fold increased risk of arteriosclerosis in patients with type 2 diabetes (T2D) (1). In the United Kingdom Prospective Diabetes Study (UKPDS), poor glycemic control in patients with T2D was associated with an increased risk of diabetes complications and, with each percentage drop in HbA1c, there was a 14% risk reduction in myocardial infarction (2). Diabetes is the leading cause for end stage renal disease (ESRD) and microalbuminuria is recognized as an independent risk factor for cardiovascular disease in patients with T2D (3). The annual rate of progression from normo-albuminuria to microalbuminuria in T2D is about 2% per year, 2.8% per year for progression from micro- to macroalbuminuria and 2.3% per year for progression from macroalbuminuria to elevated plasma creatinine or renal replacement therapy (4). As diabetic nephropathy progresses from the normoalbuminuric stage to ESRD, the annual death rate due to cardiovascular disease also increases (4).

Non-alcoholic fatty liver disease (NAFLD) is a spectrum of liver disease with high prevalence in T2D patients. The prevalence of NAFLD in T2D is 2-fold higher than in non-diabetic patients; conversely, the risk of developing T2D increases by 5-fold in patients with NAFLD (5, 6). Moreover, NAFLD can increase the risk of complications in T2D and, reciprocally, the presence of T2D in patients with NAFLD can enhance the progression to fibrosis (7). Various molecular and metabolic changes which occur in a genetically predisposed individual contribute to the pathogenesis of NAFLD; based on euglycemic clamp studies, the pathogenesis of NAFLD, and its association with insulin resistance and hyperinsulinemia, indicates that it is a feature of metabolic syndrome (8).

Although many anti-diabetic agents have been tested for efficacy against NAFLD, lifestyle modification remains the main therapeutic option, as there is no specific medication licensed for its treatment. A calorie-restricted diet along with exercise has shown histological improvement in the liver in NAFLD patients (9).

Randomized clinical studies in non-diabetic patients with thiazolidinediones, highly selective agonists for peroxisome proliferator-activated receptor gamma (PPARϒ) which sensitize adipose tissue to insulin actions and increase uptake of fatty acids in the liver, failed to show an improvement in liver enzyme levels, insulin resistance or a reduction in steatosis or inflammation. Thiazolidinediones also failed to show improvement in histological findings (10).

The glucagon-like peptide-1 (GLP-1) analogue, liraglutide, has been shown to improve insulin sensitivity in hepatocytes and adipose tissue in hyperinsulinemic euglycemic clamp studies (11). The Lifestyle, Exercise and Nutrition (LEAN) study, a 48-week randomized, double blind, placebo-controlled study in patients with non-alcoholic steatohepatitis (NASH) showed histological resolution of NASH when these patients were treated with liraglutide 1.8 mg once daily compared to placebo (12).

Sodium-glucose co-transporter 2 (SGLT-2) inhibitors are a new class of oral hypoglycemic agents used to lower blood glucose in T2D patients. These drugs have shown a reduction in oxidative stress and inflammatory markers and improved plasma levels of aminotransferase, steatosis, inflammation and fibrosis in animal models of NAFLD (13). The Effect of Empagliflozin on Liver Fat (E-Lift) trial showed that treatment with empagliflozin reduced liver fat [magnetic resonance imaging (MRI)-derived proton density fat fraction (MRI-PDFF)] and alanine transferase (ALT) levels in patients with T2D and NAFLD (14).

The current study is an observational analysis of patients with regular follow up in our diabetic clinic. We observed a high prevalence of class 1 obesity in our patients, with a mean BMI of 32.6 **±** 6 kg/m^2^ in our cohort, and prior radiological assessment was not done to exclude NAFLD or NASH. Previous epidemiological studies from Qatar have shown an overall prevalence of Metabolic Syndrome of 48.8% (15). The high prevalence of obesity and elevated ALT (5) is associated with increased risk of T2D and prompted us to look at the effect of DAPA on liver enzymes and ACR.

Our study objective was to determine the effect on liver enzymes and urine albumin-creatinine ratio (ACR) when DAPA as added-on to existing antidiabetic medications in patients with T2D. This study was done in a real-life clinic setting serving a Middle Eastern population.

## Methods

### Study Design

Observational data was collected from patients with T2D, who were initiated on DAPA 10 mg as add-on therapy to their existing antidiabetic agent from June 2017 to September 2018. All patients enrolled in this study had standard diabetes care to discuss lifestyle and diet with diabetes educators, and patients were advised to continue with their normal routine activities. A total of 40 patients were included in the study. All patients were seen in the diabetic clinic at Hamad Medical Corporation. Patient data was collected within 6-months prior to initiation of DAPA and 12-14 months post-treatment with DAPA. All patients were on established antidiabetic medications prior to initiation of DAPA therapy. In our cohort, 18 patients were taking a sulphonylurea (45%), 39 patients were taking metformin (98.0%), 26 patients were taking a DPP4 inhibitor (65.0%) and 10 patients (25%) were taking a GLP-1 agonist prior to initiation of DAPA therapy, and 2 patients were started on a GLP-1 agonist at 7 months after initiation of DAPA. Eighteen patients (45%) were on insulin therapy. Five patients were on a combination of insulin and oral triple therapy (sulphonylurea + DPP4 inhibitor + metformin and insulin). Seven patients were on a GLP-1 agonist and insulin. Eighteen patients were on a combination of an oral hypoglycemic agent (sulphonylurea + DPP4 inhibitor + metformin). Demographic and biochemical data collected included weight, BMI, HbA1c, lipid profile, alanine aminotransferase (ALT), aspartate aminotransferase (AST) and urine ACR. Patients were reviewed in clinic every 4 months and blood biochemistry was repeated at 0, 6 and 12 months. Data was collected retrospectively between June 2017 and September 2018 and, for statistical purposes, initial data (prior to initiation of DAPA) and final data at 12 months was analyzed. The primary outcome was to measure change in liver enzymes and urine ACR following initiation of DAPA as add-on therapy to existing antidiabetic medications. Secondary outcomes were reduction in hypoglycemic agents and glycosylated hemoglobin (HbA1C).

Patients were included if they were >20 years of age, had documented T2D and were newly initiated on DAPA as add-on therapy to their existing antidiabetic medications that was not changed for at least 4 months prior to DAPA initiation. Patients were excluded if they had DAPA therapy initiated prior to participation in the current study or those lost to follow up. In addition, patients with pre-existing liver disease such as hepatitis, autoimmune liver disease, alcoholic liver disease, or any known drug induced liver disease were also excluded from the study. All patients enrolled in the study denied consumption of alcohol.

### Statistical Analysis

Based on clinical observations prior to the initiation of this study, we noted that mean change in AST ranged from 7 to 10 U/L and in ALT from 3 to 7 U/L and therefore we computed sample size using effect size (changes in the mean AST is 8 U/L, statistical power 90% and level of significance 5%, therefore the sample size required was 35 participants). Descriptive statistics and Means ± Standard Deviations (SD) were calculated for all continuous variables in the study. Paired t-tests were performed to assess the mean differences and fold changes following DAPA treatment and to calculate the significance before and after the DAPA treatment. All statistical analysis was done using statistical analysis SAS version 9.4 software. A statistical significance level (P-value) of <0.05 was considered as significant.

## Results

Between June 2017 and September 2018, 40 patients with T2D were initiated on DAPA as add-on therapy to their existing antidiabetic agents to achieve better glycemic control in the study participants. Baseline characteristics are shown in **Table 1**. Mean age was 51.3 ± 9.7 years, BMI 32.3 ± 6.0, HbA1c 9.1 ± 1.1% and duration of diabetes 10.7 ± 5.4 years.

**Table 1:** Baseline measurements and whole group analysis of clinical variables pre- and post-DAPA as add-on therapy.

	Whole group analysis (n = 40).		
	Pre-DAPA/baselineMean (S.D)	Post-DAPAMean (S.D)	P
Sex (male/female)	24/16	–	
Age (Y)	51.3 (9.7)	–	
Duration of diabetes (years)	10.7 (5.4)	–	
DAPA (months of treatment)	10.2 (2.7)	–	
WT (KG)	88.7 (17.5)	85.3 (16.4)	***
BMI (kg/m2)	32.3 (6.0)	30.9 (5.4)	***
SBP (mmHg)	132.3 (16.4)	124.8 (22.4)	NS
DBP (mmHg)	75.7 (12.4)	71.0 (9.8)	NS
HbA1c (%)	9.1 (1.1)	7.4 (0.9)	***
Creatinine (µmol/L)	77.4 (15.5)	74.0 (17.9)	NS
ACR (mg/mmol)	15.0 (42.1)	11.1 (28.3)	**
TCH (mmol/L)	4.2 (0.8)	4.1 (0.8)	NS
HDL-C (mmol/L)	1.4 (2.0)	1.2 (0.4)	NS
LDL-C (mmol/L)	2.4 (0.7)	2.3 (0.7)	NS
TG (mmol/L)	1.4 (0.7)	1.4 (0.7)	NS
ALT (U/L)	43.2 (43.3)	28.9 (13.1)	***
AST (U/L)	33.9 (37.0)	23.1 (5.6)	**

The data are means and standard deviations (SD). Baseline differences, and effects of pre- and post-dapagliflozin treatment as add-on therapy. Data was assessed by paired t-test. DAPA, dapagliflozin; WT, weight; BMI, Body mass index; SBP, systolic blood pressure; DBP, diastolic blood pressure; HbA1c, hemoglobin A1c; ACR, albumin to creatinine ratio; TCH, total cholesterol; HDL-C, high density lipoprotein cholesterol; LDL-C, low density lipoprotein cholesterol; TG, triglycerides; ALT, alanine transaminase; AST, aspartate transaminase. A p value of P < 0.05 was considered significant. ***p < 0.0001, **p < 0.001. NS, non significant.

**Figure 1:**
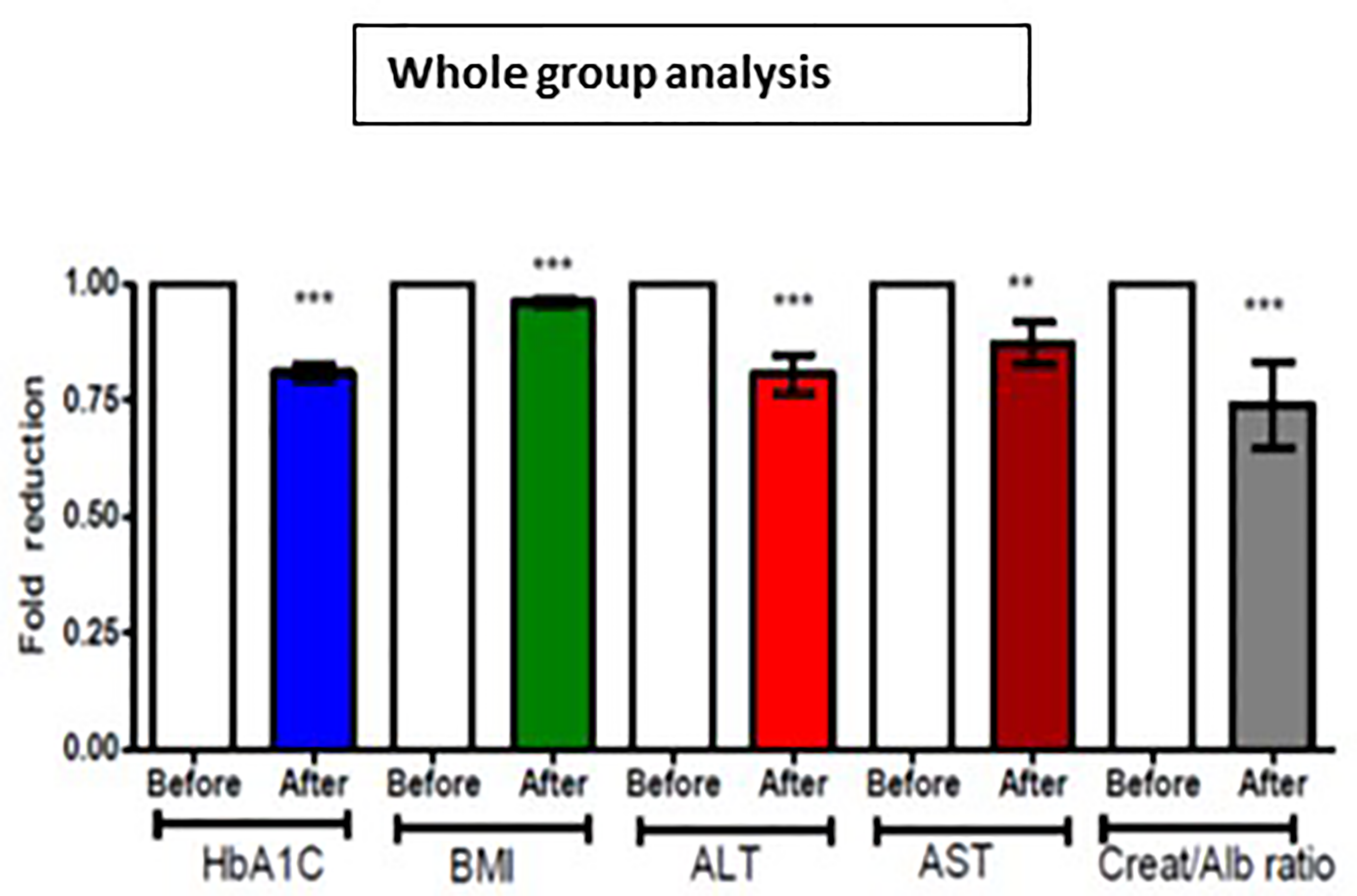
Effect of 12 months DAPA treatment as add-on therapy on HbA1c, BMI, ALT, AST and creatinine/albumin ratio whole group analysis. Significant differences were determined by paired t-test with fold difference changes following DAPA therapy **p < 0.01; ***p < 0.0001.

**Figure 2:**
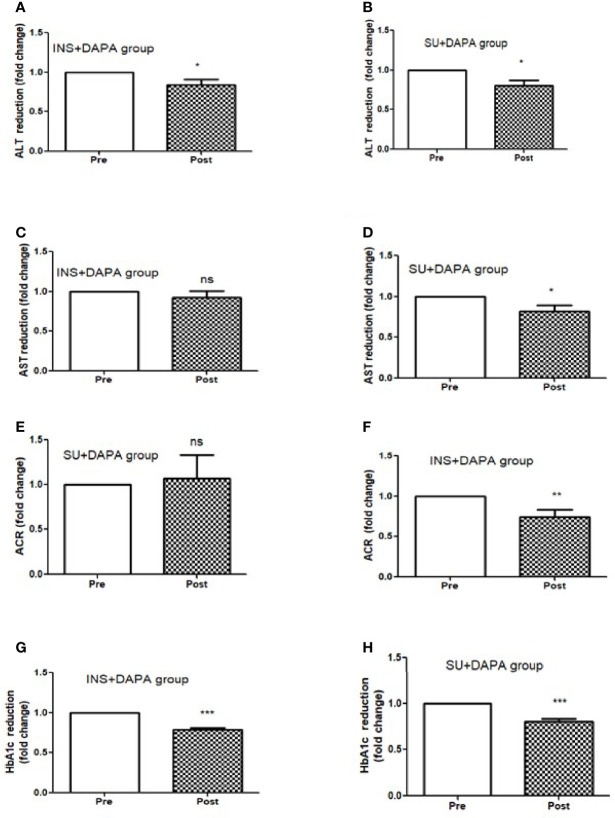
Effect of 12 months DAPA treatment as add-on therapy on ALT, AST, ACR and HbA1c subgroup analysis fold changes. **(A)** ALT reduction in insulin and DAPA group, **(B)** ALT reduction in sulfonyl and DAPA combination group, **(C)** AST reduction in insulin and DAPA group, **(D)** AST reduction in sulfonyl and DAPA combination group, **(E)** ACR reduction in insulin and DAPA group, **(F)** ACR reduction in sulfonyl and DAPA combination group. **(G)** HbA1c reduction in insulin and DAPA group, **(H)** ALT reduction in sulfonyl and DAPA combination group. Significant differences were determined by paired t-test with fold difference changes following DAPA therapy *p < 0.05; **p < 0.01; ***p < 0.0001; ns, non significant.

For the whole group, mean baseline ALT was 43.2 ± 43.3 U/l and mean AST was 33.9 ± 37.0 U/l. Following add-on therapy with DAPA, ALT and AST decreased to 28.9 ± 13.1 U/l (p<0.0001) and 23.1 ± 5.6 U/l (p<0.009), respectively. Following DAPA as add-on therapy for a duration of 12 months, there was a significant reduction in ALT (p<0.0001) and AST (p <0.009) for the whole group (**Table 1** and **Figure 1**). Eleven patients had an elevation of ALT more than 41 U/L (reference range 0-41 U/L); it was noted that these 11 patients had a mean ALT of 59.5 ± 15.6 U/L and this was reduced following DAPA treatment to 43.4 ± 12.6 U/L (P value= 0.004). Subgroup analysis of patients on insulin and DAPA showed a trend towards reduction in AST and significant reduction in ALT (**Table 2** and **Figure 2**).

**Table 2:** Pre- and post-DAPA effects of combination of insulin and DAPA on clinical variables (n = 18).

	Pre-DAPA/baselineMean (S.D)	Post-DAPAMean (S.D)	P
WT (kg)	88.27 (19.1)	84.6 (18.5)	***
BMI (kg/m^2^)	33.4 (6.8)	31.8 (6.0)	***
HbA1c (%)	9.6 (1.1)	7.6 (1.0)	***
SBP (mmHg)	129.8 (10.7)	121.6 (29.8)	NS
DBP (mmHg)	74.6 (13.2)	67.5 (9.3)	NS
ACR (mg/mmol)	19.2 (54.5)	11.3 (25.3)	***
TCH (mmol/L)	4.1 (0.7)	4.1 (0.7)	NS
HDL (mmol/L)	1.8 (2.9)	1.1 (0.3)	NS
LDL (mmol/L)	2.3 (0.7)	2.3 (0.6)	NS
TG (mmol/L)	1.7 (0.8)	1.5 (0.7)	NS
ALT (U/L)	30.9 (25.4)	14.4 (9.9)	*
AST (U/L)	24.0 (5.9)	22.9 (4.9)	NS

The data are means and standard deviations (SD). Baseline differences, and effects of combination of insulin and dapagliflozin on therapy. Data was assessed by paired t-test. WT, weight; BMI, Body mass index; SBP, systolic blood pressure; DBP, diastolic blood pressure: ACR, albumin to creatinine ratio; HbA1c, hemoglobin A1c; TCH, total cholesterol; HDL-C, high density lipoprotein cholesterol; LDL-C, low density lipoprotein cholesterol; TG, triglycerides; ALT, alanine transaminase; AST, aspartate transaminase. A p value of P < 0.05 was considered significant. *p < 0.05, ***p < 0.0001 was considered significant. NS, non significant.

Baseline mean urine ACR for the whole group (n=40) was decreased from 15.0 mg/mmol to 11.1 mg/mmol [p<0.009] following treatment with DAPA. For the whole group, the addition of DAPA to existing therapy did improve HbA1c with a reduction from 9.1 ± 1.1% to 7.4 ± 0.9% (p<0.0001). Subgroup analysis of patients who were on combination of insulin therapy and metformin therapy (n=18) showed a significant reduction in HbA1c from 9.6 ± 1.1% to 7.6 ± 1.0% (p <0.0001). Furthermore, there was a trend towards reduction in the required total daily dose of insulin in this group, although this did not reach statistical significance. Subgroup analysis of patients who were on a sulfonylurea (n=18) showed significant reductions in HbA1c (p <0.0001). After 6 months of DAPA therapy, sulphonylurea (p<0.05) was discontinued in 5 patients as they maintained good glycemic index. For the whole group, the addition of DAPA to existing therapy also showed a significant reduction in BMI (p<0.0001). Subgroup analysis showed a significant reduction in BMI for the patients on insulin therapy (p<0.0007).

**Figure 3:**
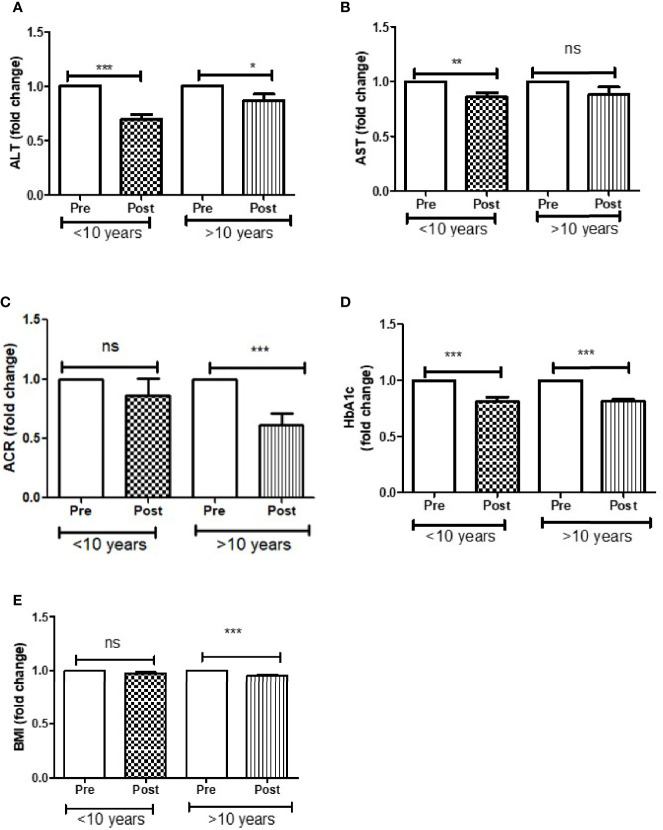
Effect of DAPA treatment as add-on therapy on ALT, AST, ACR, HbA1c and BMI were measured in T2D patients who were diagnosed with diabetes less than 10 year and more than 10 years of duration and represented as fold changes. **(A)** ALT changes, **(B)** AST changes, **(C)** ACR changes, **(D)** HbA1c changes, **(E)** BMI changes. Significant differences were determined by paired t-test with fold difference changes following DAPA therapy *p < 0.05; **p < 0.01; ***p < 0.0001; ns, non significant.

Further analysis of DAPA response in T2D patients based on duration of diabetes of less than or greater than 10 years showed that ALT reduction was greater in less than 10 years group (p<0.0001) compared to greater than 10 years group (p<0.05). AST reduction was also greater in less than 10 years group (p<0.001) compared to greater than 10 years group (ns). ACR (p<0.0001) and BMI reduction was only observed in those patients who were diagnosed with T2D more than 10 years. However, improvement in HbA1c was similar in both the groups (**Figure 3**).

## Discussion

A cross sectional epidemiological study from Qatar showed a 16% prevalence of T2D. There is an epidemic of obesity in Qatar which is closely associated with metabolic syndrome and NAFLD (15). In our study, the mean BMI was 32.3 kg/m^2^ which indicates a high prevalence of class 1 obesity associated with T2D with raised ALT. The imbalance between insulin secretion and insulin sensitivity results in hepatic insulin resistance which is well known in T2D (16). A personalized patient centered approach should be taken when initiating or optimizing therapy in patients with T2D. Newer antidiabetic medications have additional therapeutic benefits other than lowering blood sugars; these effects may be synergistic effects or may work through different pathophysiological mechanisms (17). When intensifying treatment, there is also an increased risk of hypoglycemia especially as add-on therapy to insulin or sulphonylurea. Non-insulin dependent therapies, such as SGLT-2 inhibitors, have a reduced risk of hypoglycemic incidents in T2D patients. For this reason, these medications may become a first choice, or a second choice added to metformin for treatment of T2D in the future.

In our group of patients with T2D with a mean duration of 10 years, addition of DAPA to existing anti-diabetic medications lowered ALT and AST significantly. The fold change reduction in ALT was noticeably higher in the group with duration of diabetes less than 10 years as compared to those with T2D duration of greater than 10 years. In a community based prospective study by Cho et al, the investigators found that a raised ALT level was associated with a 2-fold increase in the risk of T2D. Hepatosteatosis and insulin resistance in obese type 2 diabetes causes increased influx of fatty acid, which triggers inflammatory cytokines and hepatocyte destruction which can cause high liver enzymes (5). The effect of DAPA and other SGLT-2 inhibitors in randomized control trials has shown a disease modifying effect on patients with NAFLD in addition to its blood glucose lowering benefits. DAPA has been shown to reduce hepatocyte injury biomarkers, plasma fibroblast growth factor (FGF21), cytokeratin (CK) 18-M30 and CK 18-M65 which could explain the reduction in liver enzymes (18). Patients with adiposity-related hepatic insulin resistance have impaired beta cell dysfunction prior to diagnosis of T2D (19, 20). We observed that early initiation of DAPA as add-on therapy caused a significant reduction in ALT and a possible decrease in hepatic insulin resistance. The benefits of the addition of an SGLT-2 inhibitor were observed within the first 12 months of therapy in a real-life routine diabetic follow up clinic. Microalbuminuria in T2D is an early indicator of systemic vasculopathy, causing progressive renal damage, and is the leading cause of chronic kidney disease. In addition to the glucose lowering effects of SGLT-2 inhibitors, this class of drugs has other glucose independent effects which alter renal hemodynamics and reduce intraglomerular pressure (21).

In this study, addition of DAPA significantly reduced microalbuminuria in the whole group, though the reduction was more pronounced in patients with a duration of diabetes greater than 10 years. Most patients in our study group (82%) were already established on an ACE inhibitor prior to DAPA therapy initiation. Despite this, we observed a further reduction in microalbuminuria when DAPA was added to existing antidiabetic medications. A similar prospective randomized control trial showed a 36% reduction in 24-hour urine albumin excretion and this effect was reproduced when patients were re-exposed to DAPA 10 mg, indicating a true response to this class of antidiabetic medications (22). A more recent DAPA-CKD study showed benefit of adding DAPA to existing therapy in reducing the decline of eGFR and all-cause mortality in patients with diabetes and non-diabetic kidney disease (23).

Significant improvements in HbA1c and body weight were found when DAPA was added to existing antidiabetic medications and DAPA was well tolerated in our group of patients. Improvement in glycemic control and additional benefits of add-on therapy with DAPA were noted in patients with a duration of diabetes more than 10 years. Meta-analysis of randomized control studies in which SGLT-2 inhibitors were compared to placebo or as add-on therapy to existing medications showed long term additional benefits beyond glycemic control (24). DAPA was well tolerated when added-on to other antidiabetic medications. There was a reduction in dosage of insulin and sulphonylureas which are known to cause weight gain. No significant hypoglycemia was observed requiring either hospitalization or discontinuation of the medication. Genitourinary infections are reported as a common side effects in other large, randomized control trials with SGLT-2 inhibitors; however, in our observational study over 12 months, none of the patients reported severe genitourinary infection requiring discontinuation of the medication.

A strength of this study was that it was undertaken in a real-life clinical setting. Limitations include limited patient data capture and the small sample size. Most of the patients were on multiple medications and therefore it is not possible to eliminate the influence of the various classes of anti-diabetic medications on our study outcomes. We were aware that other medications such as GLP agonists have been shown to reduce liver enzymes. Interestingly, addition of DAPA as add-on therapy further reduced ALT and ACR. Given the nature of the study, it was not possible to eliminate all confounding factors. However, all the patients in our study were on a stable dose of medications for at least 4 months preceding the initiation of DAPA. Due to the nature of the study, we did not do radiological imaging to diagnose NAFLD or NASH prior to study initiation. However, participants in our study are likely to have undiagnosed NASH based on their risk profile and previous epidemiological studies from Qatar (15). Previous studies have already established the safety of combining DAPA as add-on therapy to other antidiabetic medications. Safety assessments after initiation of DAPA to existing antidiabetic therapy were not specifically done for this study. Patients were reviewed at 4 monthly intervals and advised to report any concerns following initiation of DAPA.

In conclusion, in a real-life clinical setting, the addition of SGLT-2 inhibitors is well tolerated and can be combined with all classes of antidiabetic medications in patients with T2D. SGLT-2 inhibitors, in addition to their benefits in improving glycemic control and weight, as add-on therapy it is associated with a reduction ACR and liver enzymes. To date, this is the first study looking at real life clinical data in a Middle Eastern T2D population.

## Data Availability Statement

The raw data supporting the conclusions of this article will be made available by the authors, without undue reservation.

## Ethics Statement 

Protocols were approved by Institutional Review Boards of the Hamad Medical Corporation, Qatar. Written informed consent for participation was not required for this study in accordance with the national legislation and the institutional requirements.

## Author Contributions

SB submitted the proposal to medical research center (MRC) at Hamad Medical Corporation and was the principle investigator for this study. MR and PC performed statistical analysis, prepared graphs, and contributed to writing the manuscript. AB researched the data and assisted with writing the manuscript. FP, IJ, JP, NM, IA, AK and SB designed the experiments, supervised progress revised and approved the final version of the article.

## Funding

This study was supported by funding received from medical research Centre, Hamad Medical corporation (MRC-01-18-322). We would like to thank medical research Centre, Hamad Medical corporation for article processing fees support.

## Conflict of Interest

The authors declare that the research was conducted in the absence of any commercial or financial relationships that could be construed as a potential conflict of interest.

## Publisher’s Note

All claims expressed in this article are solely those of the authors and do not necessarily represent those of their affiliated organizations, or those of the publisher, the editors and the reviewers. Any product that may be evaluated in this article, or claim that may be made by its manufacturer, is not guaranteed or endorsed by the publisher.

## References

[1] Kannel WB McGee DL. Diabetes and Cardiovascular Disease. The Framingham Study. JAMA (1979) 241(19):2035–8. doi: 10.1001/jama.1979.03290450033020 430798

[2] Stratton IM Adler AI Neil HA Matthews DR Manley SE Cull CA. Association of Glycaemia With Macrovascular and Microvascular Complications of Type 2 Diabetes (UKPDS 35): Prospective Observational Study. Bmj (2000) 321(7258):405–12. doi: 10.1136/bmj.321.7258.405 PMC27454 10938048

[3] Brancati FL Whelton PK Randall BL Neaton JD Stamler J Klag MJ. Risk of End-Stage Renal Disease in Diabetes Mellitus: A Prospective Cohort Study of Men Screened for MRFIT. Multiple Risk Factor Intervention Trial. JAMA (1997) 278(23):2069–74. doi: 10.1001/jama.1997.03550230045035 9403420

[4] Adler AI Stevens RJ Manley SE Bilous RW Cull CA Holman RR. Development and Progression of Nephropathy in Type 2 Diabetes: The United Kingdom Prospective Diabetes Study (UKPDS 64). Kidney Int (2003) 63(1):225–32. doi: 10.1046/j.1523-1755.2003.00712.x 12472787

[5] Cho NH Jang HC Choi SH Kim HR Lee HK Chan JC. Abnormal Liver Function Test Predicts Type 2 Diabetes: A Community-Based Prospective Study. Diabetes Care (2007) 30(10):2566–8. doi: 10.2337/dc07-0106 17626893

[6] Hazlehurst JM Woods C Marjot T Cobbold JF Tomlinson JW. Non-Alcoholic Fatty Liver Disease and Diabetes. Metabolism: Clin Exp (2016) 65(8):1096–108. doi: 10.1016/j.metabol.2016.01.001 PMC4943559 26856933

[7] Hossain N Afendy A Stepanova M Nader F Srishord M Rafiq N. Independent Predictors of Fibrosis in Patients With Nonalcoholic Fatty Liver Disease. Clin Gastroenterol Hepatol Off Clin Pract J Am Gastroenterol Assoc (2009) 7(11):1224–1229.e1221-1222. doi: 10.1016/j.cgh.2009.06.007 19559819

[8] Marchesini G Brizi M Bianchi G Tomassetti S Bugianesi E Lenzi M. Nonalcoholic Fatty Liver Disease: A Feature of the Metabolic Syndrome. Diabetes (2001) 50(8):1844–50. doi: 10.2337/diabetes.50.8.1844 11473047

[9] Vilar-Gomez E Martinez-Perez Y Calzadilla-Bertot L Torres-Gonzalez A Gra-Oramas B Gonzalez-Fabian L. Weight Loss Through Lifestyle Modification Significantly Reduces Features of Nonalcoholic Steatohepatitis. Gastroenterology (2015) 149(2):367–78.e365. doi: 10.1053/j.gastro.2015.04.005 25865049

[10] Sanyal AJ Chalasani N Kowdley KV McCullough A Diehl AM Bass NM. Pioglitazone, Vitamin E, or Placebo for Nonalcoholic Steatohepatitis. N Engl J Med (2010) 362(18):1675–85. doi: 10.1056/NEJMoa0907929 PMC2928471 20427778

[11] Armstrong MJ Hull D Guo K Barton D Hazlehurst JM Gathercole LL. Glucagon-Like Peptide 1 Decreases Lipotoxicity in Non-Alcoholic Steatohepatitis. J Hepatol (2016) 64(2):399–408. doi: 10.1016/j.jhep.2015.08.038 26394161 PMC4713865

[12] Armstrong MJ Gaunt P Aithal GP Barton D Hull D Parker R. Liraglutide Well Toleratedty and Efficacy in Patients With Non-Alcoholic Steatohepatitis (LEAN): A Multicentre, Double-Blind, Randomised, Placebo-Controlled Phase 2 Study. Lancet (2016) 387(10019):679–90. doi: 10.1016/S0140-6736(15)00803-X 26608256

[13] Tahara A Kurosaki E Yokono M Yamajuku D Kihara R Hayashizaki Y. Effects of SGLT2 Selective Inhibitor Ipragliflozin on Hyperglycemia, Hyperlipidemia, Hepatic Steatosis, Oxidative Stress, Inflammation, and Obesity in Type 2 Diabetic Mice. Eur J Pharmacol (2013) 715(1-3):246–55. doi: 10.1016/j.ejphar.2013.05.014 23707905

[14] Kuchay MS Krishan S Mishra SK Farooqui KJ Singh MK Wasir JS. Effect of Empagliflozin on Liver Fat in Patients With Type 2 Diabetes and Nonalcoholic Fatty Liver Disease: A Randomized Controlled Trial (E-LIFT Trial). Diabetes Care (2018) 41(8):1801–8. doi: 10.2337/dc18-0165 29895557

[15] Syed MA Al Nuaimi AS Latif Zainel AJA HA AQ. Prevalence of Metabolic Syndrome in Primary Health Settings in Qatar: A Cross Sectional Study. BMC Public Health (2020) 20(1):611. doi: 10.1186/s12889-020-08609-5 32362284 PMC7196222

[16] DeFronzo RA. Insulin Resistance, Lipotoxicity, Type 2 Diabetes and Atherosclerosis: The Missing Links. The Claude Bernard Lecture 2009. Diabetologia (2010) 53(7):1270–87. doi: 10.1007/s00125-010-1684-1 PMC2877338 20361178

[17] van Baar MJB van Ruiten CC Muskiet MHA van Bloemendaal L RG IJ van Raalte DH. SGLT2 Inhibitors in Combination Therapy: From Mechanisms to Clinical Considerations in Type 2 Diabetes Management. Diabetes Care (2018) 41(8):1543–56. doi: 10.2337/dc18-0588 30030256

[18] Eriksson JW Lundkvist P Jansson PA Johansson L Kvarnstrom M Moris L. Effects of Dapagliflozin and N-3 Carboxylic Acids on non-Alcoholic Fatty Liver Disease in People With Type 2 Diabetes: A Double-Blind Randomised Placebo-Controlled Study. Diabetologia (2018) 61(9):1923–34. doi: 10.1007/s00125-018-4675-2 PMC6096619 29971527

[19] Siddiqui MS Cheang KL Luketic VA Boyett S Idowu MO Patidar K. Nonalcoholic Steatohepatitis (NASH) Is Associated With a Decline in Pancreatic Beta Cell (Beta-Cell) Function. Digestive Dis Sci (2015) 60(8):2529–37. doi: 10.1007/s10620-015-3627-7 PMC4900167 25784075

[20] Cerf ME. Beta Cell Dysfunction and Insulin Resistance. Front Endocrinol (2013) 4:37. 10.3389/fendo.2013.00037 PMC3608918 23542897

[21] De Nicola L Gabbai FB Liberti ME Sagliocca A Conte G Minutolo R. Sodium/glucose Cotransporter 2 Inhibitors and Prevention of Diabetic Nephropathy: Targeting the Renal Tubule in Diabetes. Am J Kidney Dis Off J Natl Kidney Foundation (2014) 64(1):16–24. doi: 10.1053/j.ajkd.2014.02.010 24673844

[22] Petrykiv SI Laverman GD de Zeeuw D Heerspink HJL. The Albuminuria-Lowering Response to Dapagliflozin Is Variable and Reproducible Among Individual Patients. Diabetes Obes Metab (2017) 19(10):1363–70. doi: 10.1111/dom.12936 28295959

[23] Heerspink HJL Stefansson BV Correa-Rotter R Chertow GM Greene T Hou FF. Dapagliflozin in Patients With Chronic Kidney Disease. N Engl J Med (2020) 383(15):1436–46. doi: 10.1056/NEJMoa2024816 32970396

[24] Liu XY Zhang N Chen R Zhao JG Yu P. Efficacy and Well Toleratedty of Sodium-Glucose Cotransporter 2 Inhibitors in Type 2 Diabetes: A Meta-Analysis of Randomized Controlled Trials for 1 to 2years. J Diabetes Compl (2015) 29(8):1295–303. doi: 10.1016/j.jdiacomp.2015.07.011 26365905

